# Supplementary figures and images for: Short communication: Distribution of phospholipids in parotid cancer by matrix-assisted laser desorption/ionization imaging mass spectrometry
Source: PLoS One. 2021 Dec 17;16(12):e0261491. doi: 10.1371/journal.pone.0261491 (PMC8682900; doi:10.1371/journal.pone.0261491)

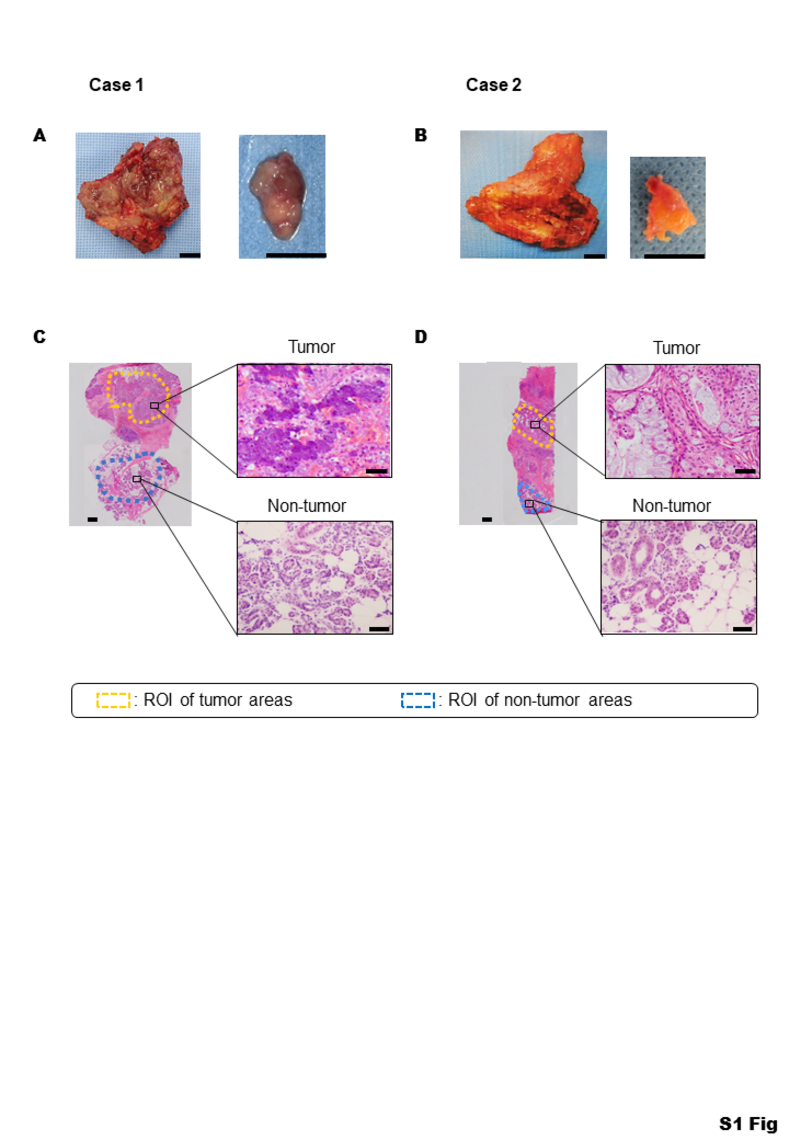

Supplement: S1 Fig — Parotid gland cancer tissue removed during surgery and cut out tissue in Cases 1 (A) and 2 (B). Scale bar: 1.0 cm. HE-stained tissue of Cases 1 (C) and 2 (D). Scale bar: 500 μm; enlarged images: 50 μm. The yellow dashed area indicates ROI of tumor areas, and the blue dashed area indicates ROI of non-tumor areas. HE, hematoxylin and eosin; ROI, region of interest. (TIF) [file pone.0261491.s001.tif]

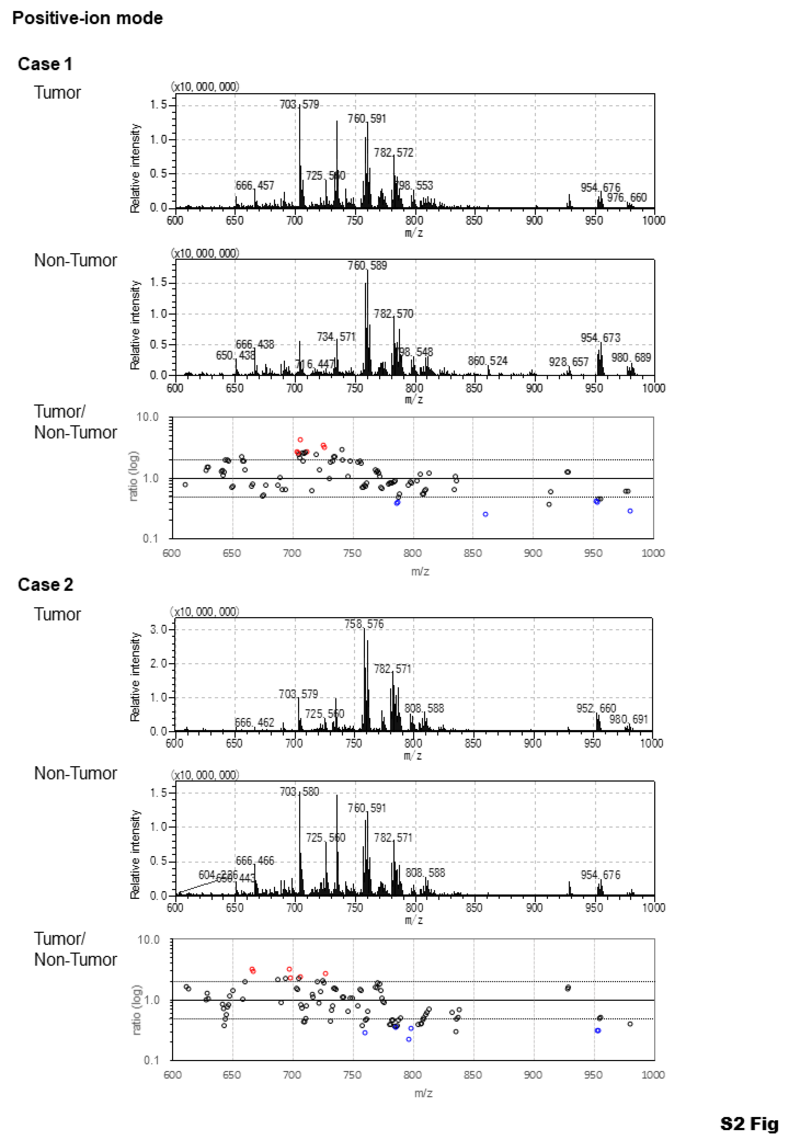

Supplement: S2 Fig — The spectra in the mass range of m/z 600–1000 in positive ion modes are shown for the tumor and non-tumor regions distinguished in S1 Fig of Case 1 and Case 2. The horizontal axis shows m/z, and the vertical axis shows the relative intensity. The dot graph of the ratio of relative intensities of tumor and non-tumor regions at each m/z is shown below. Of the peaks (m/z) with a median value of spectral intensity greater than 200, red circles indicate the top six m/z with a higher expression ratio and blue circles indicate the top six m/z with a lower expression ratio in tumor areas compared to non-tumor regions. Fig 1 shows the IMS images of each of these m/z values. (TIF) [file pone.0261491.s002.tif]

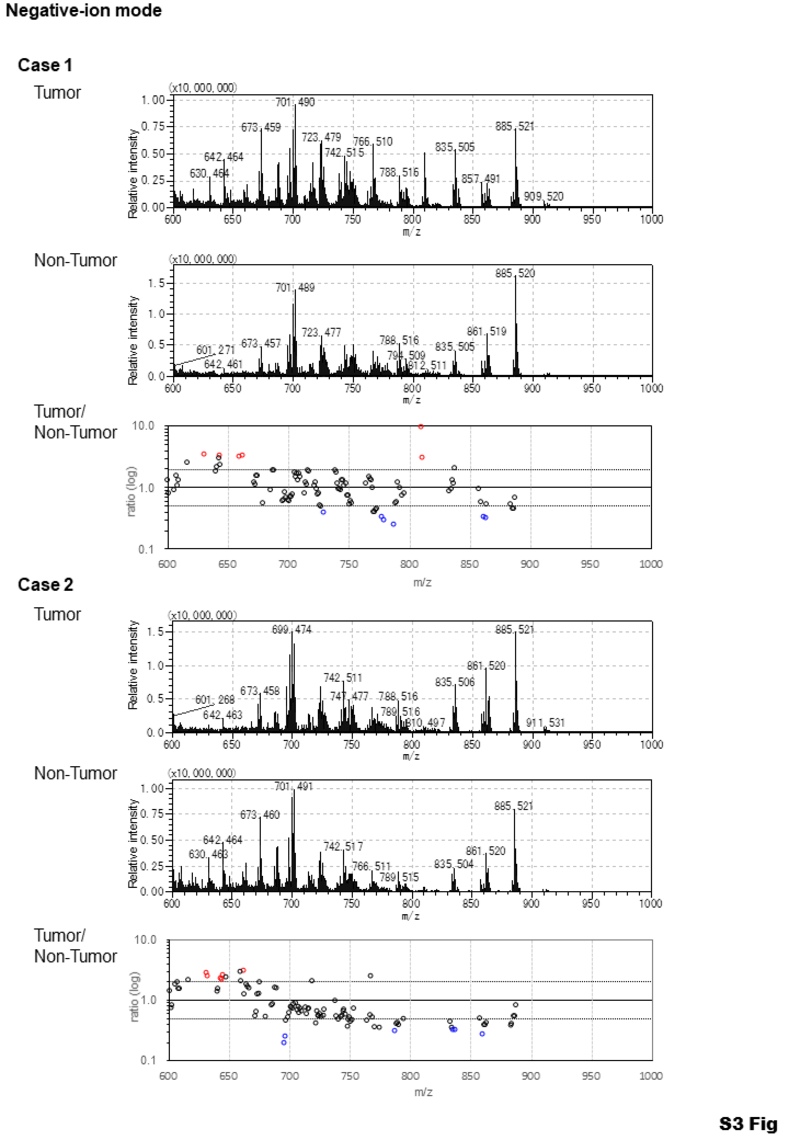

Supplement: S3 Fig — The spectra in the mass range of m/z 600–1000 in negative ion modes are shown for the tumor and non-tumor regions distinguished in S1 Fig of Case 1 and Case 2. The horizontal axis shows m/z, and the vertical axis shows the relative intensity. The dot graph of the ratio of relative intensities of tumor and non-tumor regions at each m/z is shown below. Of the peaks (m/z) with the median value of spectral intensity greater than 200, red circles indicate the top six m/z with a higher expression ratio and blue circles indicate the top six m/z with a lower expression ratio in tumor regions compared to non-tumor regions. Fig 2 shows the IMS images of each of these m/z values. (TIF) [file pone.0261491.s003.tif]
